# Complete mitochondrial genome of *Tropidophorus hangnam* (Squamata: Scincidae) with phylogenetic analysis

**DOI:** 10.1080/23802359.2020.1832928

**Published:** 2020-11-06

**Authors:** Sutee Duangjai, Suttikarn Srisodsuk, Chantip Chuaynkern, Yodchaiy Chuaynkern

**Affiliations:** a Forest Biology, Faculty of Forestry, Kasetsart University, Bangkok, Thailand; b Department of Biology, Faculty of Science, Khon Kaen University, Khon Kaen, Thailand

**Keywords:** Phylogenetic relationship, mitochondrial genome, *Tropidophorus hangnam*

## Abstract

The complete mitochondrial genome (mitogenome) of *Tropidophorus hangnam* was sequenced from its paratype (GenBank accession no. MN977920). It was 16,777 bp in length with a base composition of 31.99% A, 29.49% C, 14.34% G, and 24.18% T, and a GC content of 43.83%. The genome includes 13 protein-coding genes (PCGs), 22 transfer RNA genes, 2 ribosomal RNA genes, and a control region (D loop). Most *T*. *hangnam* genes are located on the H strand, except for the ND6 gene and eight tRNA genes, which are located on the L strand. Phylogenetic analyses based on 13 PCGs indicated that *T*. *hangnam* is sister to the clade composed of the genera *Scincella* and *Sphenomorphus*. The newly sequenced *T*. *hangnam* mitogenome will provide basic data for further studies on the genetic diversity and molecular phylogenetic relationships of the genus *Tropidophorus*.

Chuaynkern et al. (2005) first described *Tropidophorus hangnam* as a novel species. The holotype (THNHM 05776) and paratypes (THNHM 05777–85) were collected from the Phu Khaeo Wildlife Sanctuary, Chaiyaphum Province, northeastern Thailand. *Tropidophorus hangnam* is an endemic species known only from its type locality, with no additional specimens reported (Das 2010; Chanard et al. 2015); therefore, it is classified as data deficient (DD) on the International Union for Conservation of Nature (IUCN) Red List (Panitvong et al. 2018). Prior to the current work, no complete mitochondrial genome from the genus *Tropidophorus* or DNA sequences of *T*. *hangnam* were published in GenBank (https://www.ncbi.nlm.nih.gov/), and its genetic relationships with other genera of Scincidae based on mitogenome sequences are unknown. Therefore, we assembled and characterized the complete mitogenome of *T*. *hangnam* and clarified its relationships with other Scincidae genera using 13 protein-coding genes (PCG) sequences.

The paratype specimen (field no. 20502) was collected from the Phu Khaeo Wildlife Sanctuary, Chaiyaphum Province, Thailand (16°19′29′′ N 101°31′21′′ E), on 7 November 2004. Its liver tissue was fixed in 95% ethanol and stored at −20 °C. Genomic DNA was extracted from the liver tissue as described by Hillis et al. (1996), with modifications. To amplify the *T*. *hangnam* mitochondrial genome, we designed specific polymerase chain reaction (PCR) primers based on mitochondrial genome sequences obtained from *Plestiodon elegans* (KJ643142), *Scincella vandenburghi* (KU646826), and *Sphenomorphus incognitus* (MH329292). PCR primers and protocols are available upon request. The DNA sequences were assembled using the AutoAssembler version 2.1.1 software (Applied Biosystems, Foster City, CA). The assembled mitogenome was annotated using the MITOS WebServer (Bernt et al. 2013), Dual Organellar Genome Annotator (Wyman et al. 2004), and MitoFish programs (Iwasaki et al. 2013). All transfer RNA (tRNA) genes were further confirmed using the tRNAscan-SE search server (Lowe and Chan 2016).

The complete *T*. *hangnam* mitogenome was 16,777 bp in length and was deposited in GenBank (accession no. MN977920). The genomes consisted of 13 PCGs, 22 tRNA genes, 2 ribosomal RNA (rRNA) genes, and a control region (D loop). The overall base composition of the heavy strand is 31.99% A, 29.49% C, 14.34% G, and 24.18% T, with a GC content of 43.83%. Most mitochondrial genes are encoded on the heavy strand, except for *ND6* and eight tRNA genes (*tRNA^Gln^*, *tRNA^Ala^*, *tRNA^Asn^*, *tRNA^Cys^*, *tRNA^Tyr^*, *tRNA^Ser^*, *tRNA^Glu^*, and *tRNA^Pro^*) that are encoded on the light strand. Most of the 13 PCGs begin with the common initiation codon ATG, with only the *COI* gene beginning with GTG. Nine genes (*ND1*, *ND2*, *COI*, *ATPase 8*, *ATPase 6*, *ND3*, *ND4L*, *ND5*, *ND6*, and *Cytb*) end with complete stop codons (TAA, TAG, AGA, and AGG), and the other four genes (*COII*, *COIII*, *ND3*, and *ND4*) end with T as an incomplete stop codon, which is presumably completed as TAA by post-transcriptional polyadenylation (Anderson et al. 1981). The lengths of the *12S rRNA* and *16S rRNA* genes are 966 and 1519 bp, respectively. Two ribosomal subunit genes were separated by *tRNA^Val^*. The lengths of the 22 tRNA genes ranged from 65 bp (*tRNA^Cys^*) to 76 bp (*tRNA^Leu^*), and the inferred secondary structures of all tRNAs conform to the characteristic structural features of mitochondrial tRNAs. The control region (D loop) is 1380 bp in length and located between the *tRNA^Pro^* and *tRNA^Phe^* genes.

To explore the evolutionary placement of the genus *Tropidophorus* within the family Scincidae, we performed phylogenetic analyses using data from 13 PCGs of the mitogenomes of *T*. *hangnam* and 7 other skink species, with *Amblyrhynchus cristatus* (Iguanidae) as an outgroup. Phylogenetic analyses included both Bayesian inference (BI) and maximum parsimony (MP) analyses. BI was performed using the MrBayes version 3.2 software (Ronquist et al. 2012), and MP analyses were performed using the PAUP*software (Swofford 2003). Tree topologies obtained through BI and MP analyses based on the 13 PCGs were identical and statistically supported by high bootstrap and posterior probability values (Figure 1). These results indicate that *T*. *hangnam* is closer to the clade comprising *Scincella* and *Sphenomorphus* than to that comprising the genera *Isopachys* and *Plestiodon*.

**Figure 1.**
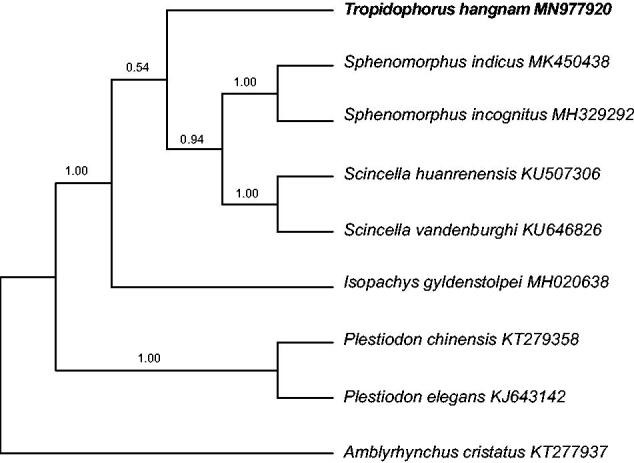
Phylogenetic tree obtained using Bayesian inference analyses from 13 protein-coding gene sequences, showing the relationship between *Tropidophorus hangnam* and other Scincidae species. Numbers above branches indicate posterior probabilities.

## Data Availability

Data supporting the findings of this study are openly available from the National Center for Biotechnology Information (NCBI; https://www.ncbi.nlm.nih.gov/) under reference no. MN977920.
